# Measuring the worldwide spread of COVID-19 using a comprehensive modeling method

**DOI:** 10.1186/s12911-023-02213-4

**Published:** 2023-09-15

**Authors:** Xiang Zhou, Xudong Ma, Sifa Gao, Yingying Ma, Jianwei Gao, Huizhen Jiang, Weiguo Zhu, Na Hong, Yun Long, Longxiang Su

**Affiliations:** 1 Department of Critical Care Medicine, State Key Laboratory for Complex Severe and Rare Diseases, Peking Union Medical College Hospital, Peking Union Medical College and Chinese Academy of Medical Sciences, Beijing, 100730 China; 2 Department of Medical Administration, National Health Commission of the People’s Republic of China, Beijing, 100044 China; 3 Digital Health China Technologies Co. Ltd, Beijing, 100080 China; 4 Department of Information Management, Peking Union Medical College Hospital, Peking Union Medical College & Chinese Academy of Medical Sciences, Beijing, 100730 China

**Keywords:** COVID-19, Group-based trajectory model, Logistic growth model, SEIR model, Trends prediction, Decision-making support

## Abstract

**Background:**

With the global spread of COVID-19, detecting high-risk countries/regions timely and dynamically is essential; therefore, we sought to develop automatic, quantitative and scalable analysis methods to observe and estimate COVID-19 spread worldwide and further generate reliable and timely decision-making support for public health management using a comprehensive modeling method based on multiple mathematical models.

**Methods:**

We collected global COVID-19 epidemic data reported from January 23 to September 30, 2020, to observe and estimate its possible spread trends. Countries were divided into three outbreak levels: high, middle, and low. Trends analysis was performed by calculating the growth rate, and then country grouping was implemented using group-based trajectory modeling on the three levels. Individual countries from each group were also chosen to further disclose the outbreak situations using two predicting models: the logistic growth model and the SEIR model.

**Results:**

All 187 observed countries' trajectory subgroups were identified using two grouping strategies: with and without population consideration. By measuring epidemic trends and predicting the epidemic size and peak of individual countries, our study found that the logistic growth model generally estimated a smaller epidemic size than the SEIR model. According to SEIR modeling, confirmed cases in each country would take an average of 9–12 months to reach the outbreak peak from the day the first case occurred. Additionally, the average number of cases at the peak time will reach approximately 10–20% of the countries’ populations, and the countries with high trends and a high predicted size must pay special attention and implement public health interventions in a timely manner.

**Conclusions:**

We demonstrated comprehensive observations and predictions of the COVID-19 outbreak in 187 countries using a comprehensive modeling method. The methods proposed in this study can measure COVID-19 development from multiple perspectives and are generalizable to other epidemic diseases. Furthermore, the methods also provide reliable and timely decision-making support for public health management.

**Supplementary Information:**

The online version contains supplementary material available at 10.1186/s12911-023-02213-4.

## Background

The global spread of COVID-19 has caused a pandemic, with cases distributed in Asia, Europe, America, Africa, Oceania and other places worldwide [1]. Although governments had implemented various measures to protect their countries/regions, such as traffic restrictions, quarantine requirements for travelers, and contact tracing, as of September 30, 2020, with the global risk continuously increasing, more than 33,774,000 cases have been confirmed in more than 180 countries, and more than 1,010,000 people have lost their lives. Related studies have revealed that COVID-19 is a highly contagious human-to-human transmission disease. The transmission rate (reproduction number range (R0)) of COVID-19 has been reported to range from 2.0 to 4.9 [2–4], which is similar to that of SARS (R0 values between 2.0 and 5.0 [5]) and higher than those of the influenza virus H1N1 (R0 values between 1.2 and 3.7 [6]) and Ebola (R0 values between 1.34 and 3.65 [7]). Although transmission was expected to decrease substantially after governments implemented various control measures, different countries exhibited different transmission control effects, and epidemic development situations remain severe.

With the number of cases growing in hundreds of countries and regions, observing and modeling the transmission dynamics and estimating the COVID-19 development globally are critical to providing decisional support for public health departments and healthcare policymakers [8]. Mathematical models were widely used in evaluating epidemic transmissions, forecasting the trend of disease spread, and providing optimal intervention strategies and control measures. A considerable number of recent studies have been conducted to estimate the scale and peak of COVID-19, and several mathematical models and prediction approaches have attempted to estimate the transmission of COVID-19 [9–14]. Among these studies, the logistic growth model and the susceptible-exposed-infected-removed (SEIR) model were the most commonly used prediction methods. A number of time series-based epidemic prediction analyses have used the logistic growth model, and the essence of the logistic model is that curve fitting and prediction results are heavily reliant on historical data. The SEIR model is a classical mathematical model for the spread of epidemics that subdivide the population into different cohorts: Susceptible (all the population are likely infected), exposed (people are exposed), infectious (people are infected), and removed (recovered). SEIR is one of the most applicable models during the early stages of epidemic, when no vaccine is available and the main control measures available are isolation of diagnosed infective cohort and social distancing. Several studies have utilized the SEIR model to estimate the transmission of COVID-19. For example, the Institute for Health Metrics and Evaluation (IHME) COVID-19 Forecasting Team modeled five COVID-19 scenarios for the United States using SEIR models [15]. Certain studies have combined the SEIR model with other methods to forecast the epidemic trends of COVID-19 in various countries, such as genetic algorithm [16]. Additionally, some studies have suggested that modifying the SEIR model can improve the prediction of COVID-19 outbreaks in particular countries, such as Spain and Italy [17]. Although these studies are all based on SEIR model, different studies often yield not exactly the same conclusions because of different data periods and parameter settings. Besides, despite the various methods and perspectives provided by previous studies on COVID-19 epidemics, the available results are still insufficient for quickly analyzing global epidemic situations and trends using a scalable framework. Additionally, in the face of a new infectious disease and its complicated features with many unknown factors, single-model estimations may infer biased results.

Therefore, to achieve objective observation and estimation of the COVID-19 outbreak and further generate reliable and timely decision-making support for public health management, we adopted a combination method based on multiple mathematical models, including the group-based trajectory modeling (GBTM), the logistic growth model and the SEIR model, to observe, analyze and predict the spread of the COVID-19 epidemic. Our comprehensive modeling methods support achieving an overall observation and comprehensive estimations of the COVID-19 outbreak in countries/regions on a large scale and these methods are generalizable to other epidemic diseases.

## Methods

### Data collection

We used reported worldwide COVID-19 epidemic data from January 23 to September 30, 2020, to observe, perform parameter estimation, and measure COVID-19 dynamics in different countries/regions. The COVID-19 epidemic data were collected from the Coronavirus COVID-19 Global Cases published by the Center for Systems Science and Engineering (CSSE) of Johns Hopkins University [18]. One hundred eighty-seven countries’ data were included for analysis.

### GBTM to identify country clusters

 As an epidemic outbreak follows the rule of rising, peaking, and then declining, classifying hundreds of countries is important to effectively observe the overall outbreak of COVID-19 globally. GBTM method was used to classify countries by their longitudinal data. Group-based trajectory modeling is mainly used to analyze longitudinal data and explore the heterogeneity in the time series objects. To compare the global outbreak situation from objective perspectives, two strategies to subgroup the 187 countries were used. First, based on each country’s daily confirmed cases over time, the probability of belonging to each potential trend group was modeled. Second, considering each country’s population, the probability of a potential trend group was modeled using the ratio of each country’s daily confirmed cases to the population as longitudinal data. Using finite mixtures of suitably defined probability distributions, group-based trajectory modeling provided a flexible and easily applied method to identify different clusters of individual case trajectories of countries and profile the characteristics of similar epidemic patterns within the clusters. In our study, a Stata plugin, traj [19, 20], was used to fit case data and model longitudinal data. Specifically, we assumed that the number of cases in the 187 countries or regions is different and there are N potential subgroups with different development patterns. We used ${Y}_{i}=({y}_{i1},{y}_{i2},\dots ,{y}_{it})$ to represent the longitudinal observation sequence value of $\mathrm{i}$ country at $\mathrm{t}$ time points, assuming the $\mathrm{t}$ components of $\mathrm{Y}\_\mathrm{i}$ obey the normal distribution. Next, we used the Gaussian mixture clustering method to divide these countries/regions into N subgroups. We tried five schemes, *n* = 2,3,4,5,6, and determined the most reasonable number of subgroups according to the Bayesian information criterion (BIC) and average posterior probability (Avepp). According to the determined number of subgroups, we performed polynomial fitting on these countries/regions to obtain the development trajectory curve of each subgroup. 

### Trends analysis of individual countries

Because countries within a cluster have similar characteristics of epidemic trends, one country from each group was randomly chosen to further disclose the growth curve and trends. Regarding the generated subgroups belonging to different levels, we randomly selected one country from each subgroup for trends analysis.

For each selected country, the average daily growth and the average daily percentage growth of confirmed cases were calculated to compare epidemic development trends in different periods. The average daily growth is calculated using the formula $\left(\mathrm{B}-\mathrm{A}\right)/\mathrm{ n}$, when the cumulative number of confirmed cases increases from $\mathrm{A}$ to $\mathrm{B}$ after $\mathrm{n}$ days. A represents the number of confirmed patients on the initial day of statistics, B represents the number of confirmed patients on the end day of statistics, and n represents the number of days between the initial and end days of statistics. The average daily percentage growth is calculated using the formula $\sqrt[n]{\frac BA}-1$, when the cumulative number of confirmed cases increases from $\mathrm{A}$ to $\mathrm{B}$ after $\mathrm{n}$ days. 

### Logistic growth model to predict the epidemic development of individual countries

First, we used a logistic growth model to observe the curves and predict the outbreaks on the individual country level. Mathematically, the logistic model describes the dynamic evolution of infected individuals controlled by the growth rate and population capacity. According to the following ordinary differential Eq. (1), we obtain the logistic function (2). The model describes the dynamic evolution of the reported number of confirmed cases P controlled by the growth rate r, and the initial value of P_0_ is the confirmed number of cases when T = 0. The maximum case volume in the environment is K, which is the limit that can be reached by increasing to the final value of $\mathrm{P}(\mathrm{t})$, and r is the growth rate. We used the least squares method to fit the logistic growth function and predict the number of future confirmed cases. Because the case numbers reported at very early stages are usually inaccurate or missing, the initial date of the model was set as the day since the 100th confirmed case was reached. We fit the logistic curve for early predictions of the peak number and growth rate of each country.

1 $$\frac{\mathrm{dP}}{\mathrm{dt}}=\mathrm{rP}(1-\frac{\mathrm{P}}{\mathrm{K}})$$

2 $$\mathrm P\left(\mathrm t\right)=\frac{KP_0e^{rt}}{K+P_0(e^{rt}-1)}$$

### SEIR model to estimate infection spread in individual countries

Based on the epidemiological characteristics of COVID-19 infection, the SEIR model is adopted because it is commonly used to study the dynamics of infectious diseases. SEIR is a deterministic metapopulation transmission model that simulates each individual in the population as a separate compartment, assuming that each individual in the same compartment has the same characteristics. By plugging in different settings of parameters, the models yield different results. In our study, we compared their results to observe patterns of COVID-19 spread.

**Fig. 1:**
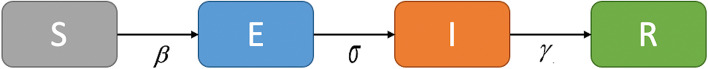
Illustration of the SEIR model and its four compartments

In the SEIR model, the population is divided into four classes: susceptible (S), exposed (E), infectious (I), and removed (R), as shown in Fig. 1. The essence of the SEIR model is a system of ordinary differential equations over time. The disease trend it predicts only depends on parameters and the start time. The model is measured by Eqs (3), (4), (5) and (6) [21], and the entire population was initially susceptible, assuming that all people have no immunity against COVID-19. The initial number of cases was collected from the reported data. To evaluate the SEIR model’s ability to predict COVID-19 infection, the data since the day that the 100th confirmed case was reached were chosen for observation, and the initial date of the model was set as the day since the 100th confirmed case was reached for each country, indicating different initial dates of the observed countries.

3 $$\mathrm{dS}/\mathrm{dt}=-\beta S\mathrm I/\mathrm N$$

4 $$\mathrm{dE}/\mathrm{dt}=\mathrm{\beta SI}/\mathrm{N}-\mathrm{\sigma E }$$

5 $$\mathrm{dI}/\mathrm{dt}=\mathrm{\sigma E}-\mathrm{\gamma I}$$

6 $$\mathrm{dR}/\mathrm{dt}=\mathrm{\gamma I}$$

$\mathrm{where}\;\mathrm S$ is the number of individuals in the susceptible population, $\mathrm{E}$ is the number of those in the exposed population, $\mathrm{I}$ is the number of those in the infected population, $\mathrm{R}$ is the number of recoveries or deaths, $\mathrm{N}=\mathrm{S}+\mathrm{E}+\mathrm{I}+\mathrm{R},$ is the number of those in the whole population, and $\upbeta$ =$\mathrm{k}*\mathrm{b}$ is the product of the people exposed to the infected population $\mathrm{k}$ and the probability of transmission $\mathrm{b}$. $\upgamma =1/\mathrm{D}$ is the average rate of recovery or death in infected populations, where $\mathrm{D}$ is the average duration of the infection, and $\upsigma$ is the rate at which exposed individuals develop into those with infections.

## Results

### Epidemic trajectory country groups

The trajectory groups of all the observed countries were generated to help disclose the global trends and clusters of countries. All the observed countries are listed in the Additional file 1: Table A.

#### GBTM results by reported daily cases

**Fig. 2:**
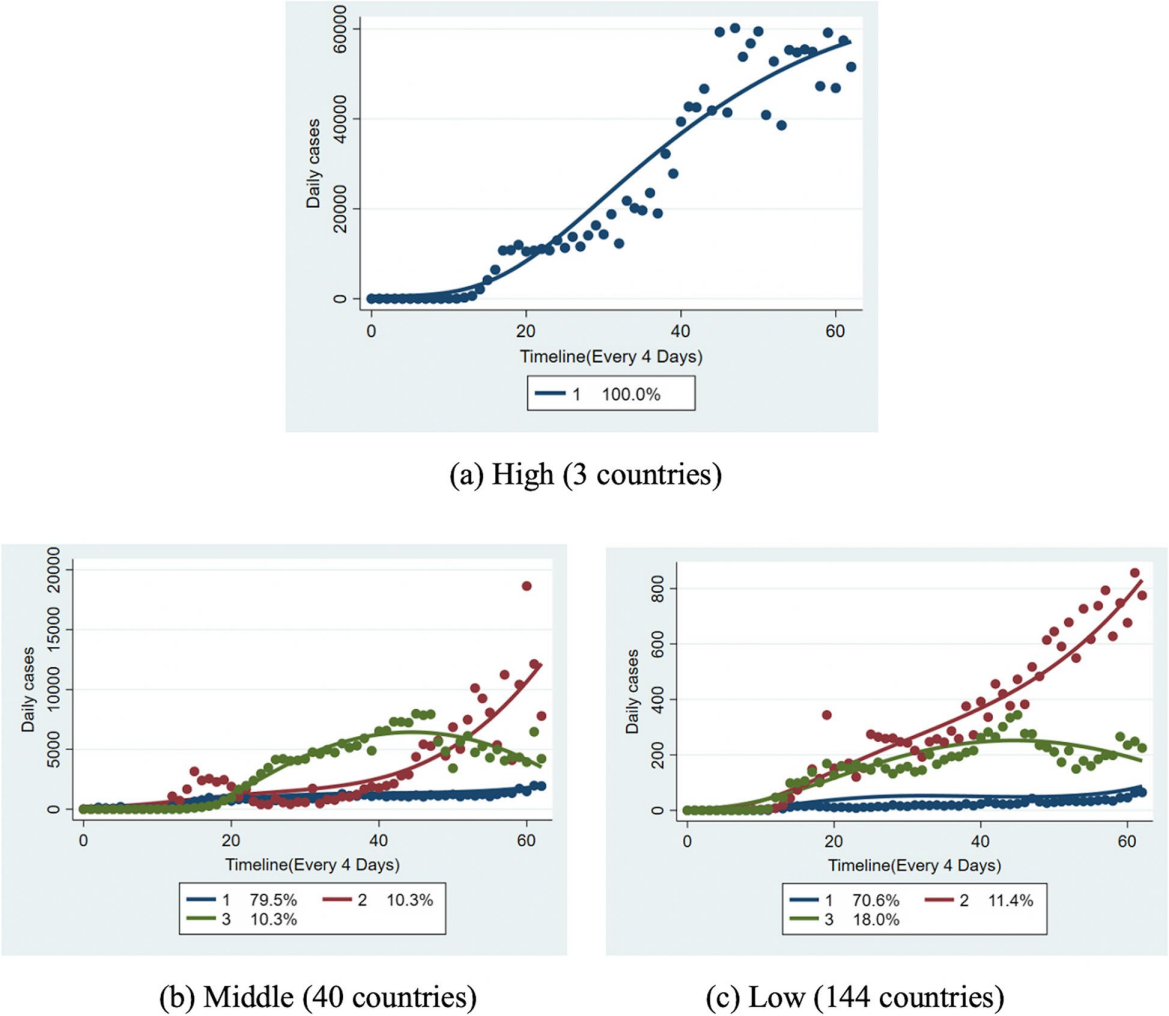
The 7 identified trajectory subgroups of the 187 observed countries by GBTM (Based on the reported number of daily cases)

According to the reported daily cases, we initially grouped the countries into three outbreak levels during the observed period. We classified the three outbreak levels as the high outbreak level group, middle outbreak level group, and low outbreak level group and then performed GBTM for each group. The output of a group-based trajectory model included group membership, estimated trajectory curves over time, and the distribution proportion for each group. As shown in Fig. 2, seven subgroups were identified from the three groups.

In high-outbreak-level countries, the daily case numbers were dramatically high, and the highest case number was beyond 60,000 in a single day during all the observation periods. One consistent trajectory group was identified by group modeling (Fig. 2a High).

Among middle-outbreak-level countries, 40 countries with the highest daily cases between 1000 and 30,000 were included, and three groups were identified by group modeling (Fig. 2b) Middle). Additionally, the country distribution proportions were displayed to compare the epidemic situations of these 40 countries. The results suggested that 10.3% of these counties (M2) had a spike above 10,000 daily cases during September 2020: 79.5% of these countries (M1) had daily cases under 2000, but some continued to show a rise; 10.3% of these countries (M3) had a slow rise in cases, with a small peak around June 2020 and then a decline thereafter. Thus, the virus spread in these countries had been effectively controlled in time.

With the inclusion criterion of a daily number of cases less than 1000, 144 countries were of low-outbreak-level. These countries were classified into three groups (Fig. 2c Low). These 144 countries had a relatively low transmission level of COVID-19, and 11.4% of them (L2) had a spike above 500 daily cases around September 2020 that continued to rise. Additionally, 18.0% of these countries (L3) have less than 300 daily cases, which had a slow rise in cases, with a small peak around June 2020 and a decline thereafter. However, the number of daily cases decreased after that and started to rise again after May 2020. The reason was likely that a series of prevention and control measures were implemented effectively but the government eased the control policies early. Furthermore, 70.6% of these countries (L1) had a stable daily number of cases that less than 100, indicating the virus spread had been contained effectively and did not evolve widely in these counties.

#### GBTM results by ratio of reported daily cases to population

**Fig. 3:**
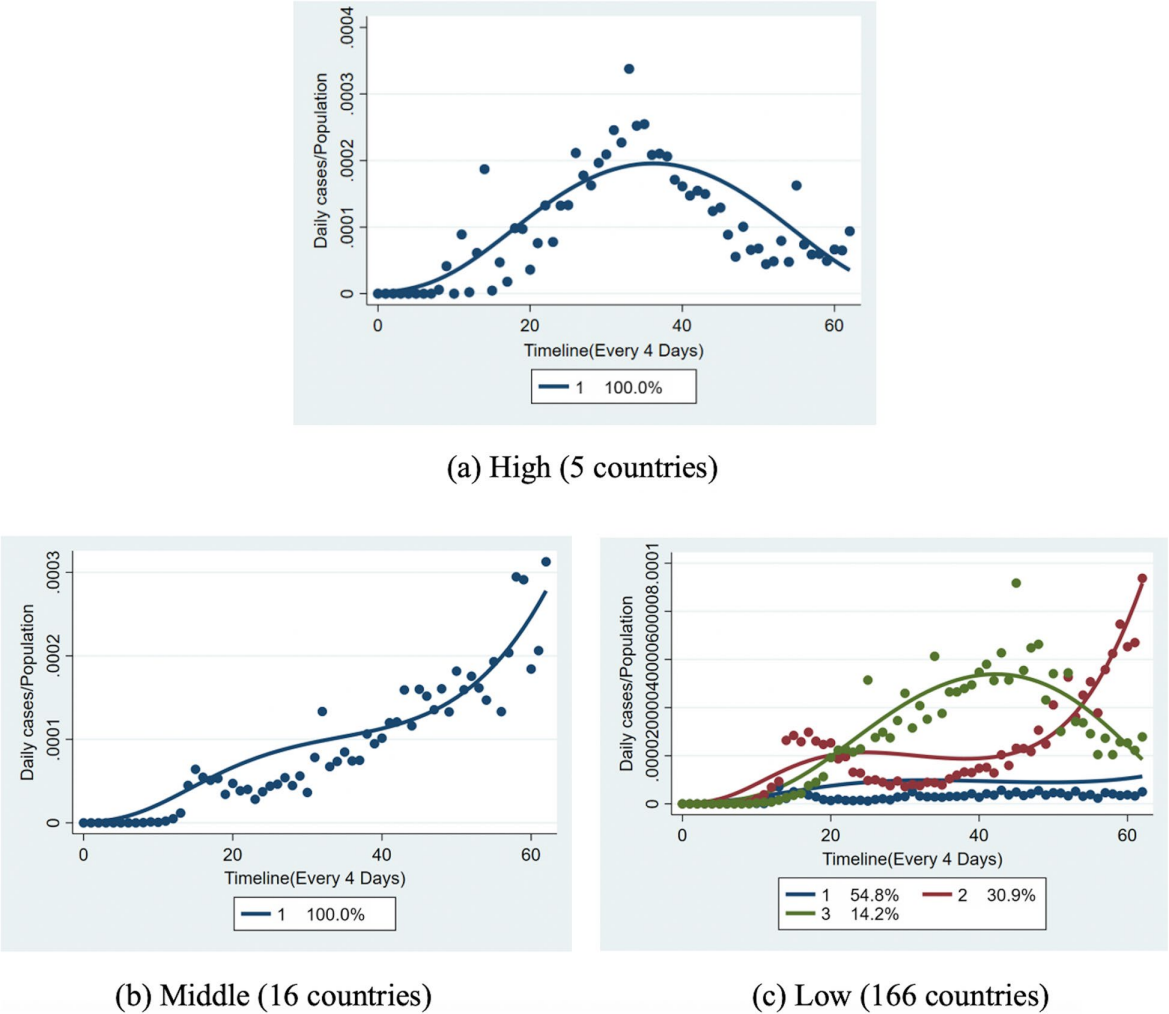
The 5 identified trajectory subgroups of the 187 observed countries by GBTM (Based on the ratio of the reported number of daily cases to population)

To consider the difference in the population size, according to the ratio of daily cases to the total population of each country and using the GBTM method, 187 countries were divided into three outbreak levels as follows: 5 high-level countries, 16 middle-level countries, and 166 low-level countries. We then grouped the countries in each outbreak level by GBTM, and five subgroups of the three outbreak levels were identified, as shown in Fig. 3.

In the 5 high-outbreak-level countries and 16 middle-outbreak-level countries, the incidence rate per capita with an epidemic development timeline was quite similar. One consistent trajectory group was identified within each level group by group modeling (Fig. 3a High and Fig. 3b) Middle).

Three subgroups were identified by group modeling among the 166 low-outbreak-level countries (Fig. 3c Low). Additionally, the country distribution proportions were displayed to compare the epidemic situations of these 166 countries. The findings were as follows: 14.2% of the countries (L1) had a spike in the incidence rate per capita around July 2020, followed by a decline; in 30.9% of the countries (L2), the incidence rate per capita increased to a small peak around April 2020, declined after that, and then increased again after August 2020; 54.8% of the countries (L1) had a low incidence rate per capita, and then their situation stabilized.

### Trends analysis of individual countries

According to two subgrouping results by GBTM, we randomly selected one country from each subgroup. For the identified 7 subgroups based on the reported number of daily cases, the average daily growth and the average daily percentage growth of confirmed cases in different periods (every quarter) are shown in Tables 1 and 2.

**Table 1:** Average daily growth of confirmed cases in different periods in selected 7 countries

Levels	Countries	The day of the first case—Mar 31	Apr 1—Jun 30	Jul 1—Sep 30
High	India	32.21	6622.45	62,724.65
Middle	Iran	1133.12	2006.79	2494.59
	Colombia	40.92	1109.27	7996.37
	Peru	50.85	3155.54	5750.45
Low	Zambia	2.43	17.54	144.25
	Poland	91.18	354.08	623.51
	Ghana	10.67	197.13	313.10

**Table 2:** Average daily percentage growth of confirmed cases in different periods in selected 7 countries (unit: %)

Levels	Countries	The day of the first case—Mar 31	Apr 1—Jun 30	Jul 1—Sep 30
High	India	13.04	6.48	2.61
Middle	Iran	27.12	1.75	0.76
	Colombia	30.75	5.14	2.33
	Peru	31.84	6.10	1.14
Low	Zambia	22.93	4.28	2.45
	Poland	32.34	2.91	1.07
	Ghana	26.10	5.11	1.04

Significant differences were observed in the average daily growth of countries in different groups, confirming the rationality of our grouping (Table 1). For the middle and low groups, the average daily growth of the three countries in different quarters has different trends; thus, they are divided into different subgroups. Because of the increase in the base number of confirmed patients, the average daily percentage of growth usually decreases gradually (Table 2). Some countries, such as Zambia, maintained a relatively high average daily percentage growth in the third quarter, although the average daily growth was not high. Public health policymakers should focus on countries with an outbreak risk in the following phases.

For the identified 5 subgroups based on the ratio of the reported number of daily cases to population, the average daily growth and average daily percentage growth in different periods (every quarter) are shown in Tables 3 and 4. Because the case data in Table 3 are not divided by the country's population, the average daily growth in these countries is not consistent with the grouping. The US is in the middle group, and the average daily growth was high in the third quarter; thus, the outbreak risk in the fourth quarter is high. Mauritania is assigned to the low group, and the average daily growth was low, so the risk of future outbreaks is not high.

**Table 3:** Average daily growth of confirmed cases in different periods in selected 5 countries

Levels	Countries	The day of the first case—Mar 31	Apr 1—Jun 30	Jul 1—Sep 30
High	Brazil	195.29	15845.24	36947.05
Middle	US	3024.01	27092.11	49999.23
Low	Iran	1133.12	2006.79	2494.59
	Syria	0.90	3.11	42.93
	Mauritania	0.28	49.08	33.30

**Table 4:** Average daily percentage growth of confirmed cases in different periods in selected 5 countries (unit: %)

Levels	Countries	The day of the first case— Mar 31	Apr 1—Jun 30	Jul 1—Sep 30
High	Brazil	28.70	6.06	1.33
Middle	US	18.61	2.80	1.10
Low	Iran	27.12	1.75	0.76
	Syria	25.89	3.78	2.97
	Mauritania	10.47	7.54	0.57

### Prediction results of individual countries

When comparing the results from the two prediction models—the logistic and SEIR models—we achieved different results for COVID-19 development predictions (Tables 5 and 6). Because different models were built on different theories and assumptions, their output measurements varied. The parameter settings and evaluation outputs were also different. For example, the cumulative number was used for the logistic model and the active number was measured by SEIR models. The results disclosed differences in these two mathematical models, but both provided an overall observation of COVID-19 development and predicted the outbreaks of representative countries from each group. In the logistic growth model, r is the growth rate, which measures the change speed of the curve, and Max is the predicted peak number of confirmed cases. In the SEIR model, four curves concerning the population of susceptible (S), exposed (E), infectious (I), and removed (R) are displayed.

**Table 5:**
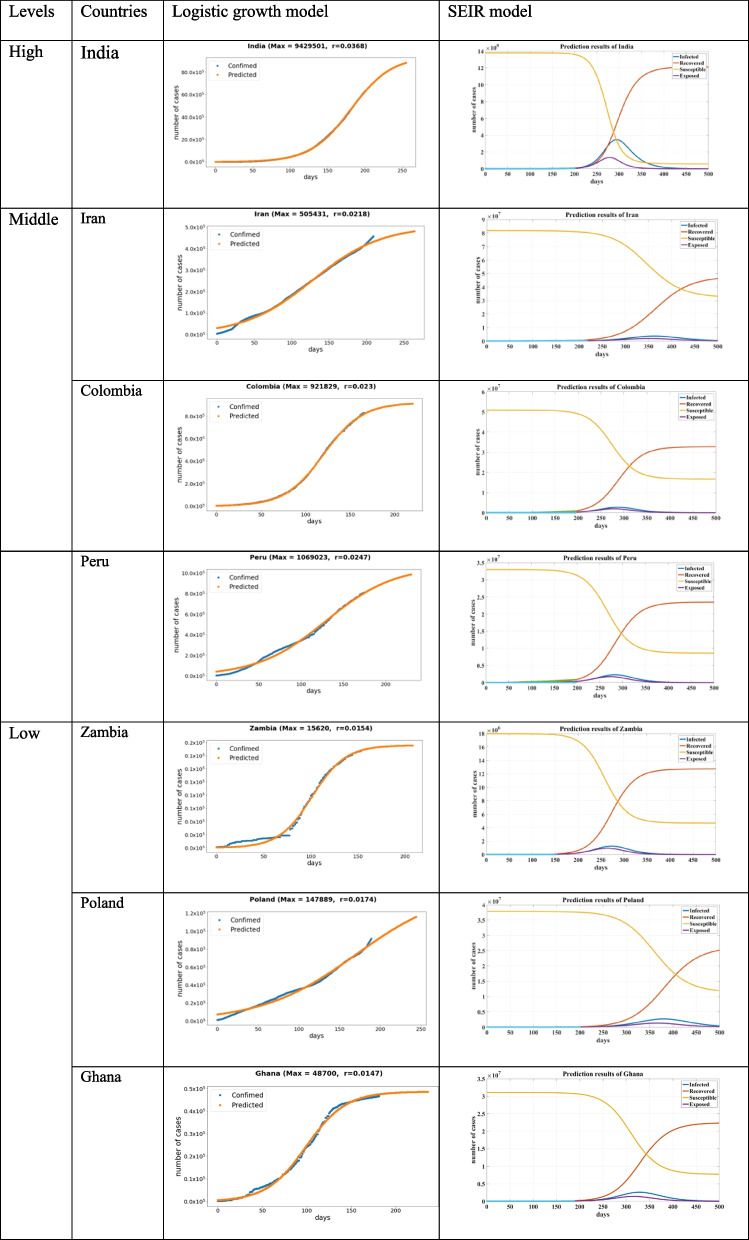
Prediction results of the logistic growth model and SEIR model (Based on subgrouping results without considering population)

**Table 6:**
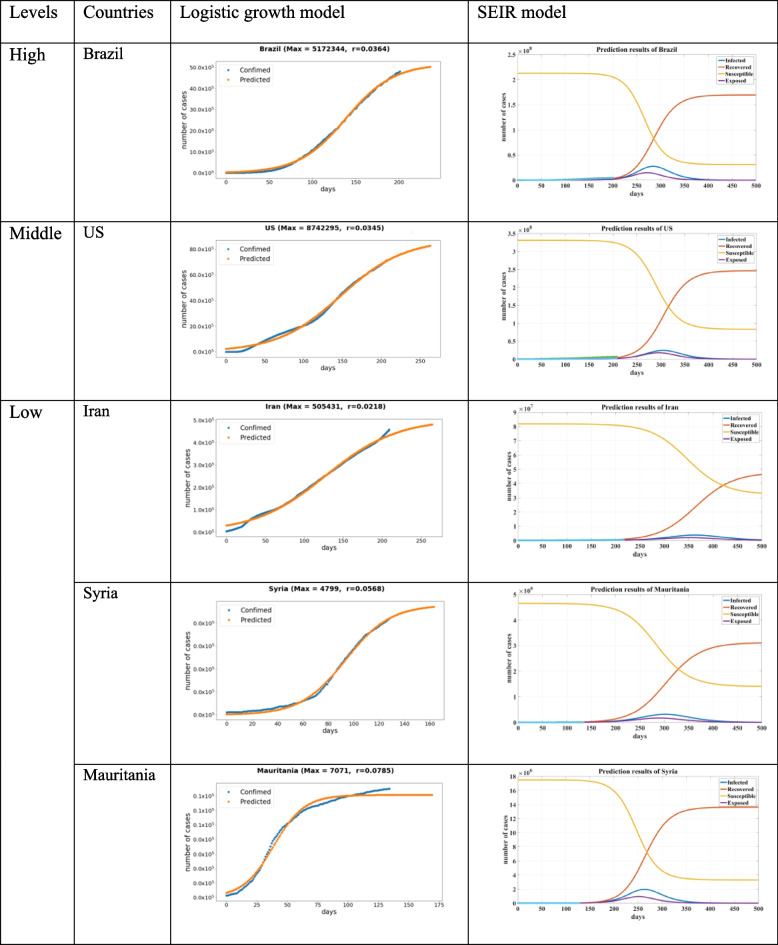
Prediction results by the logistic growth model and SEIR model (Based on subgrouping results considering population)

Table 5 lists the detailed results of the prediction trajectories of the countries selected from 7 subgroups based on our first grouping strategy (Fig. 2). Similar to the results of logistic growth modeling, India with a high outbreak level is in its rapidly rising stages but has not yet reached its peak and declining stages. Among all the predicted countries, the number of confirmed cases and the predicted peak of India are the largest. Among selected countries in the middle group, Iran (M1) is in a stage of high COVID-19 spread, and Colombia (M2) and Peru (M3) are both in a stage of a continuous rise in cases; Peru, with a higher growth rate (r), has a higher spread risk than Colombia. Furthermore, Zambia (L1), Poland (L2), and Greece (L3) are typical countries from three trajectory groups in the low outbreak level; these countries have a lower spread risk than other countries. From SEIR modeling, India was predicted to reach the highest peak number of cases of 360 million under the current rising trends. In the middle-outbreak-level countries, L1, L2, and L3 countries have a similar predicted peak size of approximately 300 thousand, and Poland (L2) had the highest outbreak prediction result, with a peak of approximately 250 thousand. Thus, SEIR modeling showed that the confirmed cases would take an average of 9–12 months to reach the outbreak peak from the day of the first case. Additionally, the active number of cases at the peak time will reach approximately 10–20% of these countries’ populations, thus overloading the healthcare system, which is the worst possible outcome.

By analyzing the predicted results of 5 selected countries (Table 6) based on our second grouping strategy (Fig. 3), we found that the number of cases in Brazil was growing rapidly and listed it in the high-level group and regarded as high risk. For this situation, control measures must be implemented in time. As the country's total population makes a critical contribution to the incidence rate per capita, considering population or not may generate different predicting results. For example, Iran was identified as the low-level group country based on subgrouping results when considering population (Table 6) but was identified as the middle-level group country based on subgrouping results without considering population (Table 5).

## Discussion

According to the reported global COVID-19 data, the development and spread of COVID-19 has been measured and predicted in our study. We compared the trends of different countries/regions using a step-by-step comprehensive method. First, GBTM was used to investigate epidemic trend differences of countries during the developmental courses of COVID-19, representing a novel attempt to apply GBTM to infectious development trajectories. Next, the growth index of trends was evaluated, and the logistic growth model and SEIR model were used to predict epidemic trends of selected individual countries from each subgroup. The grouping results of COVID-19 classified global countries into three outbreak levels and multiple trajectories subgroups, and individual countries were randomly selected for prediction modeling. Although only a small number of distinct countries were chosen for prediction after GBTM analysis, they were chosen from each subgroup and shared similar trajectory trends, making them highly representative and the comprehensive method proposed in this study allowing for being generalized and implemented in other countries/regions and other infectious diseases. Therefore, our results could reflect quantitatively an overall global situation. Using this comprehensive and step-by-step modeling method, this study measured COVID-19 development from a global perspective.

Regarding the mathematical models chosen for integration, GBTM is designed to identify clusters of individuals that follow similar trajectories of a single indicator of interest, it has been widely used in longitudinal data analysis, especially for epidemiological research [22]. Daily cases of each country were used as a single indicator to generate country clusters with similar COVID-19 outbreak trajectories. This modeling is effective to quickly and generally profiling typical trends of countries/regions on a large scale. The SEIR model is designed for infectious disease estimation; however, the logistic growth model is designed to fit the development of the curves. It has often been used in the prediction of epidemic dynamics in previous studies [9, 23]. The logistic model may fit the existing data better than the SEIR model, comparing studies using similar time window data, our logistic model shows consistent results with other studies [24]. However, it cannot be accurately evaluated and incorporates infectious characteristics. Therefore, we believe that the logistic growth model is better suited for near-term forecasts, but are incapable of characterizing long-term dynamics [25]. Instead, the SEIR model introduces more variables and factors by considering the interaction and association among multiple groups of people, and it is more reasonable than the logistic model because it follows the rules of infectious disease development. However, the prediction results vary greatly for different interventions and settings. Considering that each country has different cultures and healthcare situations leading to the implementation of the policies and control measures at different levels [26], estimating the intervention effects accurately is difficult. Therefore, our SEIR modeling was based on a macroscale perspective and provided a long-term prediction as compensation for the logistic growth model. In general, with slightly adjusting to considering new factors and different settings, our method could be used for COVID-19 or other general infectious diseases.

The study has some limitations. The mathematical models allow for the quick incorporation of multiple inputs to yield prediction results. However, this process involves making assumptions about uncertain factors. Similar to our observed results, the shape of the curve will probably change because of exogenous effects, such as the implementation of control measures and public behaviors. For example, it is difficult to determine the exact extent to which people follow the local government’s quarantine policies or measures and engage in behaviors such as washing hands, using masks, and social distancing. Furthermore, undetected transmission cases may have occurred in some countries, and sometimes the official number of cases is incomplete. When working with incomplete data, a small error in one factor can have an outsize effect. The evolution of the epidemic is complicated, and our study has only considered reported case data to implement the automatic analysis. Although we adopted an integrated method to demonstrate objective results, our model only considered the situation at the time of data collection. However, there is mounting evidence that COVID-19 development is complex and affected by multiple dynamic factors, including social activity, public health interventions, and any new situation changes; for example, with the successful development of vaccines or antiviral therapies, reduced factors need to be involved into SEIR model to keep an updated modeling solution. In the future, we will incorporate data, such as the intervention extent, intervention time, economic situation, and geographical location, by collecting, simulating, and performing automatic derivation of multidimensional data.

## Conclusions

Observation and prediction are becoming essential to infectious disease outbreak response decision-making processes. Our methods support detecting high-risk countries/regions quickly, providing reliable decision-making support for public health management dynamically, and with the ability to implement intervention policies timely for these high-risk countries, our method could help public health practitioners make early predictions, avoid healthcare systems overloading, and improve epidemic management.

## Supplementary Information

**Additional file 1: Table A.** The 187 observed countries and population.

## Data Availability

The data and codes can be downloaded from GitHub (https://github.com/Kitty0928/COVID-2019), which includes the data record of the 187 countries in the comma-separated values (CSV) format, ranging from January 23 to September 30, 2020. Based on the dataset, the coding of the GBTM, logistic model, and SEIR model was created in the Stata, Python, and Matlab mathematical computing environments.

## Abbreviations

COVID: Coronavirus Disease
SEIR: Susceptible-Exposed-Infected-Removed
SARS: Severe acute respiratory syndrome
GBTM: Group-based trajectory modeling
CSSE: Center for Systems Science and Engineering
BIC: Bayesian information criterion
Avepp: Average posterior probability

## References

[1] WHO Director-General's opening remarks at the media briefing on COVID-19 - 11 March 2020 [https://www.who.int/dg/speeches/detail/who-director-general-s-opening-remarks-at-the-media-briefing-on-covid-19---11-march-2020].

[2] Zhao S, Lin Q, Ran J, Musa SS, Yang G, Wang W, Lou Y, Gao D, Yang L, He D (2020). Preliminary estimation of the basic reproduction number of novel coronavirus (2019-nCoV) in China, from 2019 to 2020: a data-driven analysis in the early phase of the outbreak. Int J Infect Dis.

[3] Wu JT, Leung K, Leung GM (2020). Nowcasting and forecasting the potential domestic and international spread of the 2019-nCoV outbreak originating in Wuhan, China: a modelling study. Lancet.

[4] Shen M, Peng Z, Xiao Y, Zhang L. Modeling the epidemic trend of the 2019 novel coronavirus outbreak in China. The Innovation. 2020;1(3). 10.1016/j.xinn.2020.100048 PMC7831648 33521762

[5] Liu Y, Gayle AA, Wilder-Smith A, Rocklöv J (2020). The reproductive number of COVID-19 is higher compared to SARS coronavirus. J Travel Med.

[6] Boni MF, Manh BH, Thai PQ, Farrar J, Hien TT, Hien NT, Van Kinh N, Horby P (2009). Modelling the progression of pandemic influenza A (H1N1) in Vietnam and the opportunities for reassortment with other influenza viruses. BMC Med.

[7] House T (2014). Epidemiological dynamics of Ebola outbreaks. Elife.

[8] Zhou X, Ma X, Hong N, Su L, Ma Y, He J, Jiang H, Liu C, Shan G, Zhu W, Zhang S. Forecasting the worldwide spread of COVID-19 based on logistic model and SEIR model. MedRxiv. 2020:2020-03.

[9] Hermanowicz SW. Forecasting the Wuhan coronavirus (2019-nCoV) epidemics using a simple (simplistic) model. MedRxiv. 2020:2020-02.

[10] Liu T, Hu J, Kang M, Lin L, Zhong H, Xiao J, He G, Song T, Huang Q, Rong Z (2020). Transmission dynamics of 2019 novel coronavirus (2019-nCoV).

[11] Imai N, Dorigatti I, Cori A, Donnelly C, Riley S, Ferguson NM (2020). Report 2: Estimating the potential total number of novel Coronavirus cases in Wuhan City, China.

[12] Yang Z, Zeng Z, Wang K, Wong SS, Liang W, Zanin M, Liu P, Cao X, Gao Z, Mai Z, Liang J. Modified SEIR and AI prediction of the epidemics trend of COVID-19 in China under public health interventions. J Thorac Dis. 2020;12(3):165. 10.21037/jtd.2020.02.64 PMC7139011 32274081

[13] Su L, Hong N, Zhou X, He J, Ma Y, Jiang H, Han L, Chang F, Shan G, Zhu W (2020). Evaluation of the Secondary Transmission Pattern and Epidemic Prediction of COVID-19 in the Four Metropolitan Areas of China. Front Med (Lausanne).

[14] Nadler P, Wang S, Arcucci R, Yang X, Guo Y (2020). An epidemiological modelling approach for COVID-19 via data assimilation. Eur J Epidemiol.

[15] Team IC-F (2021). Modeling COVID-19 scenarios for the United States. Nat Med.

[16] Qiu Z, Sun Y, He X, Wei J, Zhou R, Bai J, Du S (2022). Application of genetic algorithm combined with improved SEIR model in predicting the epidemic trend of COVID-19, China. Sci Rep.

[17] Lopez L, Rodo X (2021). A modified SEIR model to predict the COVID-19 outbreak in Spain and Italy: Simulating control scenarios and multi-scale epidemics. Results Phys.

[18] Dong E, Du H, Gardner L (2020). An interactive web-based dashboard to track COVID-19 in real time. Lancet Infect Dis.

[19] Jones BL, Nagin DS. A note on a Stata plugin for estimating group-based trajectory models. Sociological Methods & Research. 2013;42(4):608-13.

[20] Bhavani SV, Carey KA, Gilbert ER, Afshar M, Verhoef PA, Churpek MM (2019). Identifying novel sepsis subphenotypes using temperature trajectories. Am J Respir Crit Care Med.

[21] Zou D, Wang L, Xu P, Chen J, Zhang W, Gu Q. Epidemic model guided machine learning for COVID-19 forecasts in the United States. MedRxiv. 2020:2020-05.

[22] NguenaNguefack HL, Page MG, Katz J, Choiniere M, Vanasse A, Dorais M, Samb OM, Lacasse A (2020). Trajectory modelling techniques useful to epidemiological research: a comparative narrative review of approaches. Clin Epidemiol.

[23] Pell B, Kuang Y, Viboud C, Chowell G (2018). Using phenomenological models for forecasting the 2015 Ebola challenge. Epidemics.

[24] Batista M. Estimation of a state of Corona 19 epidemic in August 2020 by multistage logistic model: a case of EU, USA, and World (Update September 2020). MedRxiv. 2020:2020-08.

[25] Wu K, Darcet D, Wang Q, Sornette D (2020). Generalized logistic growth modeling of the COVID-19 outbreak: comparing the dynamics in the 29 provinces in China and in the rest of the world. Nonlinear Dyn.

[26] Yang X, Xu T, Jia P, Xia H, Guo L, Ye K (2020). Transportation, Germs, Culture: A Dynamic Graph Model of 2019-nCoV Spread.

