# Supplementary material for: Measuring the worldwide spread of COVID-19 using a comprehensive modeling method
Source: BMC Med Inform Decis Mak. 2023 Sep 15;21(Suppl 9):384. doi: 10.1186/s12911-023-02213-4 (PMC10504693; doi:10.1186/s12911-023-02213-4)
Supplement: Supplementary file 1 — Additional file 1: Table A. The 187 observed countries and population. [file 12911_2023_2213_MOESM1_ESM.docx]

**Additional file 1**

**Table A.** The 187 observed countries and population

| **Country/Region** | **Population** |
| --- | --- |
| **Brazil** | 212559417 |
| **India** | 1380004385 |
| **US** | 331002651 |
| **Argentina** | 45195774 |
| **Bangladesh** | 164689383 |
| **Belgium** | 11589623 |
| **Bolivia** | 11673021 |
| **Canada** | 37742154 |
| **Chile** | 19116201 |
| **China** | 1439323776 |
| **Colombia** | 50882891 |
| **Czechia** | 10708981 |
| **Dominican Republic** | 10847910 |
| **Egypt** | 102334404 |
| **France** | 65273511 |
| **Germany** | 83783942 |
| **Guatemala** | 17915568 |
| **Indonesia** | 273523615 |
| **Iran** | 83992949 |
| **Iraq** | 40222493 |
| **Israel** | 8655535 |
| **Italy** | 60461826 |
| **Kazakhstan** | 18776707 |
| **Mexico** | 128932753 |
| **Morocco** | 36910560 |
| **Nepal** | 29136808 |
| **Netherlands** | 17134872 |
| **Oman** | 5106626 |
| **Pakistan** | 220892340 |
| **Panama** | 4314767 |
| **Peru** | 32971854 |
| **Philippines** | 109581078 |
| **Qatar** | 2881053 |
| **Romania** | 19237691 |
| **Russia** | 145934462 |
| **Saudi Arabia** | 34813871 |
| **South Africa** | 59308690 |
| **Spain** | 46754778 |
| **Sweden** | 10099265 |
| **Turkey** | 84339067 |
| **Ukraine** | 43733762 |
| **United Kingdom** | 67886011 |
| **Afghanistan** | 38928346 |
| **Albania** | 2877797 |
| **Algeria** | 43851044 |
| **Andorra** | 77265 |
| **Angola** | 32866272 |
| **Antigua and Barbuda** | 97929 |
| **Armenia** | 2963243 |
| **Australia** | 25499884 |
| **Austria** | 9006398 |
| **Azerbaijan** | 10139177 |
| **Bahamas** | 393244 |
| **Bahrain** | 1701575 |
| **Barbados** | 287375 |
| **Belarus** | 9449323 |
| **Belize** | 397628 |
| **Benin** | 12123200 |
| **Bhutan** | 771608 |
| **Bosnia and Herzegovina** | 3280819 |
| **Botswana** | 2351627 |
| **Brunei** | 437479 |
| **Bulgaria** | 6948445 |
| **Burkina Faso** | 20903273 |
| **Burma** | 54409800 |
| **Burundi** | 11890784 |
| **Cabo Verde** | 555987 |
| **Cambodia** | 16718965 |
| **Cameroon** | 26545863 |
| **Central African Republic** | 4829767 |
| **Chad** | 16425864 |
| **Comoros** | 869601 |
| **Congo (Brazzaville)** | 5518087 |
| **Congo (Kinshasa)** | 89561403 |
| **Costa Rica** | 5094118 |
| **Cote d'Ivoire** | 26378274 |
| **Croatia** | 4105267 |
| **Cuba** | 11326616 |
| **Cyprus** | 1207359 |
| **Denmark** | 5792202 |
| **Djibouti** | 988000 |
| **Dominica** | 71986 |
| **Ecuador** | 17643054 |
| **El Salvador** | 6486205 |
| **Equatorial Guinea** | 1402985 |
| **Eritrea** | 3546421 |
| **Estonia** | 1326535 |
| **Eswatini** | 1160164 |
| **Ethiopia** | 114963588 |
| **Fiji** | 896445 |
| **Finland** | 5540720 |
| **Gabon** | 2225734 |
| **Gambia** | 2416668 |
| **Georgia** | 3989167 |
| **Ghana** | 31072940 |
| **Greece** | 10423054 |
| **Grenada** | 112523 |
| **Guinea** | 13132795 |
| **Guinea-Bissau** | 1968001 |
| **Guyana** | 786552 |
| **Haiti** | 11402528 |
| **Holy See** | 801 |
| **Honduras** | 9904607 |
| **Hungary** | 9660351 |
| **Iceland** | 341243 |
| **Ireland** | 4937786 |
| **Jamaica** | 2961167 |
| **Japan** | 126476461 |
| **Jordan** | 10203134 |
| **Kenya** | 53771296 |
| **Kosovo** | 2000700 |
| **Kuwait** | 4270571 |
| **Kyrgyzstan** | 6524195 |
| **Laos** | 7275560 |
| **Latvia** | 1886198 |
| **Lebanon** | 6825445 |
| **Lesotho** | 2142249 |
| **Liberia** | 5057681 |
| **Libya** | 6871292 |
| **Liechtenstein** | 38128 |
| **Lithuania** | 2722289 |
| **Luxembourg** | 625978 |
| **Madagascar** | 27691018 |
| **Malawi** | 19129952 |
| **Malaysia** | 32365999 |
| **Maldives** | 540544 |
| **Mali** | 20250833 |
| **Malta** | 441543 |
| **Mauritania** | 4649658 |
| **Mauritius** | 1271768 |
| **Moldova** | 4033963 |
| **Monaco** | 39242 |
| **Mongolia** | 3278290 |
| **Montenegro** | 628066 |
| **Mozambique** | 31255435 |
| **Namibia** | 2540905 |
| **New Zealand** | 4822233 |
| **Nicaragua** | 6624554 |
| **Niger** | 24206644 |
| **Nigeria** | 206139589 |
| **North Macedonia** | 2083374 |
| **Norway** | 5421241 |
| **Papua New Guinea** | 8947024 |
| **Paraguay** | 7132538 |
| **Poland** | 37846611 |
| **Portugal** | 10196709 |
| **Rwanda** | 12952218 |
| **Saint Kitts and Nevis** | 53199 |
| **Saint Lucia** | 183627 |
| **Saint Vincent and the Grenadines** | 110940 |
| **San Marino** | 33931 |
| **Sao Tome and Principe** | 219159 |
| **Senegal** | 16743927 |
| **Serbia** | 8737371 |
| **Seychelles** | 98347 |
| **Sierra Leone** | 7976983 |
| **Singapore** | 5850342 |
| **Slovakia** | 5459642 |
| **Slovenia** | 2078938 |
| **Somalia** | 15893222 |
| **Sri Lanka** | 21413249 |
| **Sudan** | 43849260 |
| **Suriname** | 586632 |
| **Switzerland** | 8654622 |
| **Syria** | 17500658 |
| **Tajikistan** | 9537645 |
| **Tanzania** | 59734218 |
| **Thailand** | 69799978 |
| **Timor-Leste** | 1318445 |
| **Togo** | 8278724 |
| **Trinidad and Tobago** | 1399488 |
| **Tunisia** | 11818619 |
| **Uganda** | 45741007 |
| **United Arab Emirates** | 9890402 |
| **Uruguay** | 3473730 |
| **Uzbekistan** | 33469203 |
| **Venezuela** | 28435940 |
| **Vietnam** | 97338579 |
| **West Bank and Gaza** | 5101414 |
| **Western Sahara** | 597339 |
| **Yemen** | 29825964 |
| **Zambia** | 18383955 |
| **Zimbabwe** | 14862924 |
